# The role of pre-existing cross-reactive antibodies in determining the efficacy of vaccination in humans: study protocol for a randomized controlled trial

**DOI:** 10.1186/s13063-015-0651-z

**Published:** 2015-04-10

**Authors:** Jenny G Low, Limin Wijaya, Greg KY Li, Eleanor YL Lim, Aland KL Shum, Yin-Bun Cheung, Eng-Eong Ooi

**Affiliations:** Department of Infectious Diseases, Singapore General Hospital, 20 College Road, Singapore, Singapore 169856; Singhealth Investigational Medicine Unit, Singapore General Hospital, Outram Road, Singapore, Singapore 169608; Centre for Quantitative Medicine, Duke-NUS Graduate Medical School, 8 College Road, Singapore, Singapore 169857; Program in Emerging Infectious Diseases, Duke-NUS Graduate Medical School, 8 College Road, Singapore, Singapore 169857

**Keywords:** Live vaccination, Cross-reactive neutralizing antibodies, Innate immune response, Adaptive immune response

## Abstract

**Background:**

Epidemic viral diseases have become more prevalent.. Among the various strategies to prevent such epidemics, vaccination is the most cost-effective. However, populations that are immunized are typically already exposed to multiple previous vaccinations or natural infections. Studies from this and other laboratories have revealed that pre-existing dengue antibodies can either inhibit or enhance subsequent dengue infection depending on the pre-existing antibody levels. While cross-reactive antibody is potentially pathogenic in dengue, how it impacts immune response to vaccination is unclear. Aggregated at the site of vaccination and the respective draining lymph nodes are antigen-presenting and immune regulatory cells that express Fc receptors and play pivotal roles in determining the magnitude and polarity of the immune response. Vaccine uptake by these antigen-presenting cells may thus be either inhibited or enhanced when vaccines are opsonized with cross-reactive antibodies.

**Design:**

In view of the limited knowledge on how cross-reactive antibodies affect vaccination outcome, we propose a study that exploits the known cross reactivity between Japanese encephalitis (JE) virus antibody and yellow fever (YF) vaccine. We hypothesize that cross-reactive antibodies impact antibody response to YF at the point vaccination in a concentration-dependent manner by altering both vaccine uptake and the innate immune response by antigen presenting cells. We will structure an open-label clinical trial on sequential vaccination with JE and YF vaccines, with different time intervals between vaccinations. This would test immune response to YF vaccination in subjects with different titer of cross-reactive JE vaccine-derived antibodies. The clinical materials obtained in the trial will drive basic laboratory investigations directed at elucidating how heterologous antibody affect vaccination at the molecular level. YF neutralizing antibody titer will be measured using plaque reduction neutralization test against the vaccine strain YF17D. Innate immune response will be characterized genetically using either microarray or digital PCR (or both). The innate immune response will also be characterized at the protein and metabolite level using Luminex bead technology and lipidomic/metabolomic approaches.

**Discussion:**

This proposed study represents one of the first to examine the role of cross-reactive antibodies in modulating immune responses to vaccines, the findings of which may re-shape vaccination strategy.

**Trial registration:**

Clinical Trials.gov registration number: NCT01943305 (3 September 2013).

## Background

The increasing prevalence of viral epidemics in recent decades threatens both human health and global economies. Among the countermeasures, vaccination remains the single most cost-effective method of disease prevention. One of the most popular forms of vaccines is the live attenuated vaccine (LAV). LAV is a weakened virus that is able to mimic natural infection and present antigens in native conformation to immune cells, often resulting in superior protection compared to other forms of vaccines. However, populations that are immunized are typically already exposed to multiple previous vaccinations or natural infections against a range of viruses. Since some of these viruses are evolutionarily related and share antigenic epitopes with the LAV, there is high likelihood of cross-reactivity between LAV and pre-existing antibodies evoked against previous vaccination or infection. Although the impact of these cross-reacting antibodies has largely been overlooked, there is growing evidence that its impact can be highly significant but widely varied [1-3]. Thus, cross-reactive antibodies could, in some cases, boost the efficacy of vaccines while in others render them ineffective.

Studies from this and other laboratories have revealed that pre-existing antibodies against certain dengue virus (DENV) serotypes can enhance subsequent infection with a heterologous serotype by promoting viral entry and infection into Fc receptor-expressing cells [4-7]. While the presence of cross-reactive antibodies is potentially detrimental in dengue [5,8-11], it is unclear how cross-reactive antibodies may impact the efficacy of LAV or other viral vector-based vaccines. Some of the critical sites in the body where cross-reactive antibodies could impact vaccination efficacy are at the site of vaccination [12,13] and in the secondary lymph node draining the vaccination site, where the innate and adaptive immune responses are initiated, respectively [14,15]. Aggregated at these sites are dendritic cells, monocytes, macrophages, and mast cells, which are either antigen presenting or immune regulatory cells that play pivotal roles in determining the magnitude and polarity of the immune response. As all of these cell types express Fc receptor, cross-reactive antibodies can potentially and markedly alter the nature of the initial interactions of vaccine antigens with these immune surveillance and regulatory cells and, by extension, the resulting immune response [16]. It is conceivable that cross-reactive antibodies may directly bind vaccine antigen and enhance Fc receptor uptake by antigen presenting cells resulting in an enhanced and beneficial immune response. Alternatively, these antibodies may aggregate vaccines to co-ligate inhibitory Fc receptors, a mechanism that we recently demonstrated [17], that reduces both entry into these cells and hence vaccination efficacy. This capacity of pre-existing antibodies to interfere with efficacy of vaccines may explain why the use of adenovirus and reovirus to deliver antigens has encountered very mixed results [2,3,18-21].

### Objectives and hypotheses

In view of the limited information available on the potentially critical role heterologous antibodies have in modulating the efficacy of LAVs, we propose here a study that takes advantage of the known cross-reactivity between antibodies to Japanese encephalitis (JE) virus and yellow fever (YF) vaccine. We will structure human and animal studies that inform on whether heterologous antibody affects vaccination efficacy and how this is mediated molecularly. Our long-term goal is to maximize the cost-effectiveness of vaccination by developing a personalized approach to vaccination where the dose of vaccine is tailored to the background heterologous antibody titer of the vaccinee.

We hypothesize that cross-reactive antibodies impact antibody response to YF at the point vaccination in a concentration-dependent manner by altering both vaccine uptake and the innate immune response by antigen-presenting cells.

Primary objectives:

- The primary objective of this clinical study is to examine the role of cross-reactive antibodies in modulating immune responses to vaccines.
- Hypothesis: antibody response to YF vaccination is influenced by the heterologous antibody titer at the point of YF vaccination.
- Hypothesis: heterologous antibody to YF affects the magnitude of vaccine uptake through the Fc receptors and alters the quality and magnitude of the innate immune response following YF vaccination, which has been previously shown to be predictive of the immune response to YF vaccination.
- Hypothesis: heterologous antibody titer affects the response of Fc receptor-expressing antigen presenting or immune regulatory cells to YF vaccination.

Secondary objectives:

1. To determine neutralizing antibody response to YF vaccination in human volunteers at different time intervals following a prior JE vaccination.
2. To characterize the innate immune response to YF vaccination at different time intervals after a prior JE vaccination.
3. To elucidate the dendritic cell, mast cell, monocyte and macrophage response to YF vaccination in the presence of heterologous JE antibodies in humans.

Exploratory objectives:

1. To examine how heterologous antibody titer influences the antibody response to vaccination. While an obvious approach is to enroll participants with varying titers of pre-existing antibodies, such as dengue antibodies, that cross-react with YF vaccine this would require a large population size to account for qualitative and quantitative differences. To overcome this we will take advantage of the natural reduction in cross-reactive antibodies with increasing amount of time from a prior JE vaccination.
2. To maximize the cost-effectiveness of vaccination by developing a personalized approach to vaccination where the dose of vaccine is tailored to the background heterologous antibody titer of the vaccine.

## Methods/design

### Trial approval and conduct

The trial is approved by the Singhealth centralised Institutional Review Board (ID: 2013/385/E) and is registered on the clinicaltrials.gov registration number: NCT01943305. The trial sponsor is the Singapore General Hospital, Singapore, in collaboration with Duke-NUS Graduate Medical School, Singapore. The trial is funded by the Biomedical Research Council, Singapore (BMRC reference 13/1/96/19/689) and will be carried out in accordance with the principles of the Singapore Good Clinical Practice guidelines and in compliance with the Helsinki Declaration. Informed consent is obtained for all patients before enrolment.

### Study design

The study design is shown in Table 1. Healthy volunteers meeting all inclusion criteria will be enrolled and recruited into the study at the Singhealth Investigational Medicine Unit (IMU).

**Table 1:** **Study design**

**Groups**	**Vaccination schedule**		
	**1st vaccination (month 1)**	**2nd vaccination (month 2)**	**3rd vaccination**
Group 1	JE vaccination	JE vaccination	Yellow fever vaccination 1 month after second JE vaccine
Group 2	JE vaccination	JE vaccination	Yellow fever vaccination 4 months after second JE vaccine
Group 3	JE vaccination	JE vaccination	Yellow fever vaccination 9 months after second JE vaccine
Group 4	Yellow fever vaccination	No vaccination	No vaccination

JE, Japanese encephalitis.

A total of 100 healthy adults, pre-screened to be negative for anti-dengue antibodies, will be enrolled upon written informed consent; 75 subjects in the test arm will receive two doses of inactivated JE vaccine (Ixiaro, Novartis (Singapore) Pte Ltd, Pharmaceutical Division 20 Pasir Panjang Road #10-25/28 Mapletree Business City Singapore 117439), while 25 subjects in the control arm will receive no JE vaccination.

Subjects in the test arm will be sub-divided into three groups who receive YF17D (Stamaril, SANOFI PASTEUR SA 2, avenue Pont Pasteur F-69007 Lyon France): group 1, YF vaccination 1 month post-JE vaccination; group 2, YF vaccination 4 months post-JE vaccination; group 3, YF vaccination 9 months post-JE vaccination. Subjects in the control arm (group 4) will receive YF vaccination at month 1.

Blood sampling in relation to YF17D vaccination will take place: 1) pre-dose; 2) 1 day post-dose; 3) 3 days post-dose (+1 day); 4) 1 week post-dose (±2 days); 5) 1 month post-dose (±5 days); and 6) 6 months post-dose (±14 days).

### Selection and withdrawal of patients

#### Inclusion criteria

- Healthy male or female adults, 21 to 50 years of age at the time of screening.
- Negative for anti-dengue antibodies by enzyme-linked immunosorbent assay (ELISA).
- Subjects who are willing to comply with the requirements of the study protocol and scheduled visits (for example, completion of the subject diary, return for follow-up visits) and who are willing to make themselves available for the duration of the study with access to a consistent means of telephone contact, which may be either at home or at the workplace, and be either a land line, or mobile, but not a pay phone or other multiple-user device (that is, a common-use phone serving multiple rooms or apartments).
- Subjects who give written informed consent approved by the Ethical Review Board governing the site.
- Satisfactory baseline medical assessment as assessed by physical examination and a stable health status. The laboratory values must be within the normal range of the assessing site or show abnormalities that are deemed not clinically significant as judged by the investigator. A stable health status is defined as the absence of a health event satisfying the definition of a serious adverse event.
- Accessible vein in the forearm for blood collection.
- Females of non-child bearing potential due to surgical sterilization (hysterectomy or bilateral oophorectomy or tubal ligation) or menopause.
- Female subjects of childbearing potential may be enrolled in the study
- if they have a negative urine pregnancy test on the day of screening and negative urine dipstick pregnancy tests at the days of the vaccinations
- if they use adequate, reliable contraception or abstain from sexual intercourse during the entire study for 1 year.

#### Exclusion criteria

1. Presence of acute infection in the preceding 7 days or presence of a temperature ≥38.0 °C (oral temperature assessment), or acute symptoms greater than “mild” severity on the scheduled date of first vaccination.
2. History of severe drug and/or food allergies and/or known allergies to the trial product or its components.
3. Any condition that, in the opinion of the investigator, would complicate or compromise the study or wellbeing of the subject.
4. Woman who are pregnant or breast feeding.
5. History or presence of cardiovascular, respiratory, hepatic, renal, gastrointestinal, neuropsychiatric, or immunosuppressive disorders that would be a risk factor when administering the investigational product.
6. History of thymus gland disease.
7. Diagnosed with cancer or receiving treatment for cancer within the 3 years prior to the screening.
8. Evidence of clinically significant anemia and other any significant active hematological disease, or having donated >450 mL blood within the past 3 months.
9. Evidence of substance abuse, or previous substance abuse.
10. Participation in a study involving administration of an investigational compound within the past 4 months, or planned participation during the duration of this study.
11. Administration of any licensed vaccine, such as MMR and/or chickenpox immunization within 30 days before the first study vaccine dose.

#### Premature withdrawal of patients from study

Participants are free to withdraw consent and discontinue participation at any time without penalty and are not obliged to give a reason. Subjects may be withdrawn/removed, if necessary, to protect their health or the integrity of the study. The investigator also has the right to withdraw participants from the study in the event of inter-current illness, adverse events, treatment failures, protocol violations, administrative, or other reasons. When a subject withdraws from the study, all safety data normally required at the completion of the study will be obtained, where possible. All details available will be reported and recorded for any subject that withdraws or is removed from the study.

The Principal Investigator and/or the Sponsor of this study may stop a subject’s participation in the study at any time for one or more of the following reasons:

- Failure to follow the instructions of the Principal Investigator and/or study staff.
- The Principal Investigator decides that continuing participation could be harmful.
- Treatment is needed that is not allowed in the study.
- The study is cancelled.
- Other administrative reasons.
- Unanticipated circumstances.

### Dosing regimen

**Figure 1:**
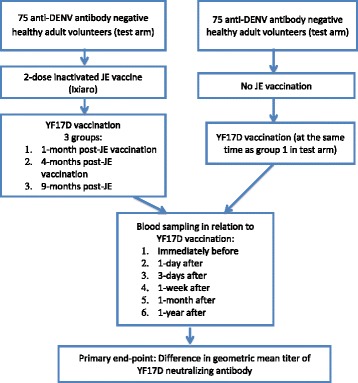
Dosing regimen. DENV, dengue virus; JE, Japanese encephalitis; YF, yellow fever.

The dosing regimen is shown in Figure 1.

### Methods and assessments

Subjects will be recruited via the Singhealth IMU healthy volunteer database. Eligible subjects will be approached and invited for participation. The participants will be given the participant information sheet and allowed to ask any questions about the trial. The Principal Investigator or a Co-investigator will take informed consent. Participants meeting all inclusion criteria and no exclusion criteria and who agree to participate will be enrolled and randomized at the IMU.

#### Visit 1 – screening for all groups

Screening procedures will commence once a subject has given informed consent. A physical examination and blood samples will be taken for baseline assessments: dengue immunoglobulin G specific for previous dengue infection, and hematology for full blood count and clinical chemistry (liver and renal panel). Urine will be collected for a pregnancy test (for female subjects of child-bearing potential only).

The study screening procedure is for all subjects, regardless of their randomized group:

- Informed consent
- Physical examination
- Urine pregnancy test if applicable
- Safety bloods which include:
- Full Blood Count (FBC)
- Dengue IgG
- Liver panel
- Renal panel

Subjects must receive the first dose of vaccination within 21 days of the screening visit.

#### Procedures for groups 1, 2 and 3

The following procedures for subjects randomized to groups 1, 2 and 3 are conducted at the following visits.

#### Visit 2 – dose 1 (Japanese encephalitis)

- Pre-dose vital signs
- Urine pregnancy test if applicable
- JE vaccination
- 1 hour post-dose physical examination
- 1 hour post-dose vital signs

#### Visit 3 – dose 2 (Japanese encephalitis)

This visit will occur 1 month after the first JE vaccination.

- Pre-dose vital signs
- JE vaccination
- 1 hour post-dose physical examination
- 1 hour post-dose vital signs

#### Visit 4 – dose 3 (yellow fever)

This visit will occur 1 month after the second JE vaccination for subjects randomized to group 1, 4 months after the second JE vaccination for subjects randomized to group 2, or 9 months after the second JE vaccination for subjects randomized to group 3.

- Pre-dose vital signs
- Pre-dose research blood for YF antibody immune response and RNA
- YF vaccination
- 1 hour post-dose vital signs
- 1 hour post-dose physical examination

#### Visit 5 – 1 day after yellow fever vaccination

- Vital signs
- Physical examination
- Research blood for YF antibody immune response and RNA
- Assessment of clinical symptoms

#### Visit 6 – 3 days (+1 day) after yellow fever vaccination

- Vital signs
- Physical examination
- Research blood for YF antibody immune response and RNA
- Assessment of clinical symptoms

#### Visit 7 – 1 week (±2 days) after yellow fever vaccination

- Vital signs
- Physical examination
- Research blood for YF antibody immune response
- Assessment of clinical symptoms

#### Visit 8 – 15 days (+2 days) after yellow fever vaccination

- Vital signs
- Physical examination
- Assessment of clinical symptoms
- Review subject diary
- Review adverse events and concomitant medications

#### Visit 9 – 1 month (±5 days) after yellow fever vaccination

- Vital signs
- Physical examination
- Research blood for YF antibody immune response

#### Visit 10 – 6 months (±14 days) after yellow fever vaccination

- Vital signs
- Physical examination
- Research blood for YF antibody immune response

#### Procedures for group 4

The following procedures for subjects randomized to group 4 are conducted at these visits.

#### Visit 2 – Dose 1 (yellow fever)

- Pre-dose vital signs
- Urine pregnancy test if applicable
- Pre-dose research blood for YF antibody immune response and RNA
- YF vaccination
- 1 hour post-dose vital signs
- 1 hour post-dose physical examination

#### Visit 3 – 1 day after yellow fever vaccination

- Vital signs
- Physical examination
- Research blood for YF antibody immune response and RNA
- Assessment of clinical symptoms

#### Visit 4 – 3 days (+1 day) after yellow fever vaccination

- Vital signs
- Physical examination
- Research blood for YF antibody immune response and RNA
- Assessment of clinical symptoms

#### Visit 5 – 1 week (±2 days) after yellow fever vaccination

- Vital signs
- Physical examination
- Research blood for YF antibody immune response
- Assessment of clinical symptoms

#### Visit 6 – 15 days (+2 days) after yellow fever vaccination

- Vital signs
- Physical examination
- Assessment of clinical symptoms
- Review subject diary
- Review adverse events and concomitant medications

#### Visit 7 – 1 month (±5 days) after yellow fever vaccination

- Vital signs
- Physical examination
- Research blood for YF antibody immune response

#### Visit 8 – 6 months (±14 days) after yellow fever vaccination

- Vital signs
- Physical examination
- Research blood for YF antibody immune response

#### Further assessments

During each study visit, subjects will document all symptoms they may experience after the vaccination with YF vaccine. These assessments include local symptoms as well as systemic symptoms. Investigators will check on the presence of fever, nausea, vomiting and myalgia at each point at day 1, day 3, week 1 and day 15. Patients will be monitored again at month 1 and month 6. The patient and their diary will be assessed and reviewed by the investigators to ensure that there are no other symptoms experienced by the subjects. Subjects will be trained to watch out for important clinical signs and symptoms and any unusual manifestations such as high fever, unusual behavior, fits, yellowing of skin and eyes, or any signs and symptoms of a serious allergic reaction such as difficulty breathing, hoarseness or wheezing, hives, paleness, weakness, a fast heart beat or dizziness. They will be educated to inform the study site personnel and the Principal Investigator should any of them develop any of these features.

YF neutralizing antibody titer will be measured using a plaque reduction neutralization test against the vaccine strain YF17D. Innate immune response will be characterized genetically using either microarray or digital PCR (or both). The innate immune response will also be characterized at the protein and metabolite level using Luminex bead technology and lipidomic/metabolomic approaches.

Schematic diagrams of the study assessments are show in Tables 2, 3, 4 and 5.

**Table 2:** **Study schema for study arms 1, 2 and 3**

	**Visit 1 screening (-21 to -1 days)**	**Visit 2 - first vaccination (day 0)**		Visit 3 - second vaccination (+2 weeks from first vaccination)*		Visit 4 - third vaccination (+2 weeks from second vaccination)*	
		**Pre-vaccination**	**1 hour post-vaccination**	**Pre-vaccination**	**1 hour post-vaccination**	**Pre-vaccination**	**1 hour post-vaccination**
Inform consent	×						
Medical history and demographics	×						
Physical examination	×	x	x	x	x		×
Vital signs^a^	×	×	×	×	×	×	×
Hematology labs^b^	×						
Chemistry labs (liver, renal panel^c^)	×						
Dengue immunoglobulinG^d^	×						
Urine pregnancy test^e^	×	×					
Adverse events^f^	×	×		×		×	
Concomitant medications	×	×		×		×	
Blood sampling in relation to YF17D vaccination						×^g^	

*Window period: for second vaccination is +2 weeks (vaccination cannot be done earlier); for third vaccination (group 1, 2, 3) is +2 weeks (vaccination cannot be done earlier). ^a^Vital signs include temperature (oral and tympanic allowed), blood pressure, pulse rate, and respiratory rate. ^b^Hematology labs include hemoglobin, red blood cell, white blood cell, hematocrit, platelet count, neutrophil, lymphocyte, monocyte, eosinophil, basophil. ^c^Liver panel includes total protein, albumin, total bilirubin, alkaline phosphatase, ALT alanine transaminase, AST aspartate transaminase, gamma-glutamyl transpeptidase. Renal panel includes urea, sodium, potassium, chloride, bicarbonate, creatinine. ^d^Dengue immunoglobulin G will be tested by enzyme-linked immunosorbent assay. ^e^For females only. ^f^Clinical Trial Certificate Adverse Events version 4.0 will be used for grading. ^g^For third vaccination, pre-dose blood sample can be taken any time before dosing. YF, yellow fever.

**Table 3:** **Blood sampling schedule for study arms 1, 2, 3**

	**Visit 4**		**Visit 5 - 1 day after**	**Visit 6 - 3 days after (+1 day)**	**Visit 7 - 1 week after (±2 days)**	**Visit 8 - 15 days after (+2 days)**	**Visit 9 - 1 month after (±5 days)**	**Visit 10 - 6 months after (±14 days)**
	**Pre-vaccination (any time before dosing)**	**Post-vaccination (1 hour)**						
Physical examination		×	×	×	×	x	×	×
Vital signs^a^	×	×	×	×	×	x	×	×
Assessment for clinical symptoms (local and general symptoms)		×	×	×	×	x		
Blood sampling in relation to YF17D vaccination	×		×	×	×		×	×
Adverse events^b^	×		×	×	×	x	×	×
Concomitant medications	×		×	×	×	x	×	×

^a^Vital signs include temperature (oral and tympanic allowed), blood pressure, pulse rate, and respiratory rate. ^b^Clinical Trial Certificate Adverse Events version 4.0 will be used for grading. YF, yellow fever.

**Table 4:** **Schedule in relation to YF17D vaccination**

	**Visit 4**		**Visit 5 - 1 day after**	**Visit 6 - 3 days after (±1 day)**	**Visit 7 - 1 week after (±2 days)**	**Visit 8 - 1 month after (±5 days)**	**Visit 9 - 1 year after (±7 days)**
	**Pre-vaccination (any time before dosing)**	**Post-vaccination (1 hour)**					
Physical examination		×	×	×	×	×	×
Vital signs^a^	×	×	×	×	×	×	×
Assessment for clinical symptoms (local and general symptoms)		×	×	×	×	×	
Blood sampling in relation to YF17D vaccination	×		×	×	×	×	×
Adverse events^b^	×		×	×	×	×	×
Concomitant medications	×		×	×	×	×	×

^a^Vital signs include temperature (oral and tympanic allowed), blood pressure, pulse rate, and respiratory rate. ^b^Clinical Trial Certificate Adverse Events version 4.0 will be used for grading. YF, yellow fever.

**Table 5:** **Study schema for study group 4**

	**Visit 1 – screening (-21 to -1 days)**	**Visit 2 - first vaccination (day 0)**		**Visit 3 - 1 day after**	**Visit 4 - 3 days after (+1 day)**	**Visit 5 - 1 week after (±2 days)**	**Visit 6 - 15 days after (+2 days)**	**Visit 7 - 1 month after (±5 days)**	**Visit 8 - 6 months after (±14 days)**
		**Pre-vaccination**	**Post-vaccination (1 hour)**						
Inform consent	×								
Medical history and demographics	×								
Physical examination	×		×	×	×	×	x	×	×
Vital signs^a^	×	×	×	×	×	×	x	×	×
Haematology labs^b^	×								
Chemistry labs (liver, renal panel^c^)	×								
Dengue immunoglobulin G^d^	×								
Urine pregnancy test^e^	×	×							
Adverse events^f^	×	×		×			x		
Concomitant medications	×	×		×			x		
Blood sampling in relation to YF17D vaccination		×^g^		×	×	×		×	×
Assessment for clinical symptoms (local and general symptoms)			×	×	×	×	x		

^a^Vital signs include temperature (oral and tympanic allowed), blood pressure, pulse rate, and respiratory rate. ^b^Hematology labs include hemoglobin, red blood cell, white blood cell, hematocrit, platelet count, neutrophil, lymphocyte, monocyte, eosinophil, basophil. ^c^Liver panel includes total protein, albumin, total bilirubin, alkaline phosphatase, ALT alanine transaminase, AST aspartate transaminase, gamma-glutamyl transpeptidase. Renal panel includes urea, sodium, potassium, chloride, bicarbonate, creatinine. ^d^Dengue immunoglobulin G will be tested by enzyme-linked immunosorbent assay. ^e^For females only. ^f^Clinical Trial Certificate Adverse Events version 4.0 will be used for grading. ^g^For third vaccination, pre-dose blood sample can be taken any time before dosing. YF, yellow fever.

#### Sample size calculation and statistical analysis

The main analysis will be comparisons between each of the JE groups and the no JE group. To allow for the risk of inflated type 1 error rate, a Bonferroni-type adjustment will be used. A sample size of 21 per group gives 80% power (5%/3 = 1.7% two-sided type 1 error) to detect a difference of 1 unit of log(2)-transformed antibody titer between two groups, assuming the standard deviation is 1 on the log(2) scale. We anticipate a drop-out rate of approximately 20%. However, given the long duration between JE and YF vaccination in group 3 of the JE vaccination arm a higher loss to follow-up rate could occur in this group. Given that group 2 in the JE vaccination arm serves as an intermediate while the largest difference in YF antibody titers is hypothesized to be in groups 1 and 3 in the JE vaccination arm, we weighted each arm differently. Consequently, n = 25 in the YF control arm, n = 25 for the group that receives YF vaccination 1 month after JE vaccination, n = 22 for the group that receives YF vaccination 4 months after JE vaccination, and n = 28 for the group that receives YF vaccination 9 months after JE vaccination. The total number of participants is therefore 100.

#### Randomization

To reduce potential bias in this open-label trial, subjects will be randomized to the four treatment arms (groups 1, 2, 3 and 4); 100 sealed envelopes, each containing a study arm, will be randomly picked by a non-study team member (akin to drawing lots).

#### Data management

Direct data capture of demographic and clinical data will be conducted using computer notebooks. The data will be kept confidential in password-protected computers accessible only by selected research staff. Identifiers will be kept in a separate file in another office and every effort will be made to protect the privacy of the participants. The data to be analyzed will contain only de-identified data. An electronic data capture system will be used.

### Safety assessment

#### Definitions

An adverse event (AE) is defined as any untoward medical occurrence in a subject administered a medicinal product, which does not necessarily have a causal relationship with this treatment.

A serious adverse event (SAE) is defined as any untoward medical occurrence that at any dose is life-threatening, requires inpatient hospitalization or prolongation of existing hospitalization, results in persistent or significant disability/incapacity or is a congenital anomaly/birth defect.

For AEs, event terminology, the date and time of event start and end, severity, relatedness, impact to the continuation of the study, and final outcome of the event will be recorded on the case report form from day 0 to the last study visit until the event has been resolved. For a SAE, in addition to the above, an event summary, the criteria used to categorize the event as an SAE, and a list of all tests and treatments given for the event will be documented.

#### Serious adverse event reporting

SAEs require expedited reporting. SAEs, whether or not they are thought to be related to the investigational drug, must be reported to the study monitor within 24 hours. Within that time, a completed SAE worksheet and other available supporting documentation must be forwarded to the study monitor by facsimile to JGL. All patients with SAEs will be followed for outcome.

For SAEs that are related and unexpected resulting in death or life-threatening events, an initial report must be made to the Singhealth Institutional Review Board and the Clinical Trials Branch of the Health Sciences Authority within 7 calendar days of the event. For SAEs that are related and unexpected resulting in non-fatal or non-life threatening events, notification must occur within 15 calendar days of the event. Refer to the Health Science Authority’s Guidance “Safety Reporting Requirements for Clinical Trial Drugs” for further details of reporting requirements.

#### Study monitoring

The study may be evaluated by government inspectors/regulatory authorities who must be allowed access to electronic case report forms, source documents, and other study files. The inspectors will review case report forms and compare them with source documents to verify accurate and complete collection of data and confirm that the study is being conducted according to the protocol, Singapore Good Clinical Practices and all applicable regulations.

## Discussion

As far as we are aware, this is the first proof-of-concept trial assessing the role of cross-reactive neutralizing antibody in subsequent vaccinations. If results from this study suggest the immune response to vaccination is modulated by pre-existing cross-reactive neutralizing antibodies, this will potentially re-shape vaccination strategy.

## Trial status

The trial has been granted a Clinical Trial Certificate 707 by the Health Sciences Authority (certificate number: CTC 1300291). The trial is currently still actively recruiting and is anticipated to be completed by June 2015.

## Abbreviations

AE: Adverse event
DENV: Dengue virus
ELISA: Enzyme-linked immunosorbent assay
IMU: Investigational medicine unit
JE: Japanese encephalitis
LAV: Live attenuated vaccine
SAE: Serious adverse event
YF: Yellow fever
